# Association of Initial Laxative Strategy With Clinical Outcomes in Critically Ill Patients With Acute Myocardial Infarction: A Retrospective Cohort Study

**DOI:** 10.1155/cdr/1649836

**Published:** 2026-04-11

**Authors:** Bingfu Wang, Yulong Song, Yujian Fan, Zhiqiang Zhao

**Affiliations:** ^1^ Department of Cardiovascular Medicine, The First Affiliated Hospital of Henan University of Chinese Medicine, Zhengzhou, China, hactcm.edu.cn; ^2^ First Teaching Hospital of Tianjin University of Traditional Chinese Medicine, Tianjin, China, tjtcm.cn; ^3^ National Clinical Research Center for Chinese Medicine, Tianjin, China

**Keywords:** acute myocardial infarction, bowel management, clinical outcomes, laxative strategy, retrospective cohort study

## Abstract

**Background:**

Although laxatives are commonly used for constipation in critically ill patients with acute myocardial infarction (AMI), the optimal initial choice is unclear. We are aimed at comparing the clinical outcomes of initiating therapy with stimulant, stool softener, and osmotic laxatives.

**Methods:**

This retrospective study analyzed ICU patients with AMI from the MIMIC‐IV database, comparing three initial laxative strategies using propensity score matching (PSM). The primary outcome was 28‐day mortality. Secondary outcomes included ICU, in‐hospital, and 365‐day mortality; cardiogenic shock; malignant arrhythmia; delirium; and bowel sound recovery. Findings were validated with inverse probability of treatment weighting (IPTW) and subgroup analyses.

**Results:**

In the 1:1:1 PSM cohort of 1887 patients, stool softeners were associated with significantly lower mortality versus stimulants: in‐hospital (adjusted OR = 0.39, 95% CI 0.22–0.69, p = 0.001), 28‐day (aHR = 0.55, 95% CI 0.40–0.76, p < 0.001), and 365‐day (aHR = 0.57, 95% CI 0.45–0.72, p < 0.001). Osmotic laxatives correlated with lower in‐hospital (aOR = 0.52, 95% CI 0.30–0.89, p = 0.017) and 28‐day (aHR = 0.72, 95% CI 0.55–0.95, p = 0.019) mortality, but not 365‐day compared with stimulant laxatives. Both agents were linked to lower delirium risk and better bowel sound recovery. IPTW sensitivity analyses showed consistent results.

**Conclusion:**

Among ICU‐admitted AMI patients, the initial use of stool softeners or osmotic laxatives was associated with a lower risk of short‐term mortality compared with stimulant laxatives. These findings indicate that a more robust and gentle laxative management strategy may have potential clinical value for this high‐risk population.

## 1. Introduction

Acute myocardial infarction (AMI) is a leading cause of morbidity and mortality worldwide and a significant public health threat. According to the Heart Disease and Stroke Statistics—2024 Update, an estimated 605,000 new cases of AMI occur annually in the United States [1, 2]. Due to its acute onset and severity, admission to the intensive care unit (ICU) for close monitoring and life support is a cornerstone of AMI management. In the United States, approximately 35%–60% of AMI patients are admitted to the ICU or other critical care settings, with in‐hospital mortality rates ranging from 4% to 7% [3, 4].

In the ICU, patients with AMI are prone to constipation due to treatments (e.g., fluid restriction and diuretics) and pathophysiological features such as sympathetic overactivation and prolonged bed rest [5]. Regarding acute triggers, the Valsalva maneuver during defecation causes drastic fluctuations in intrathoracic pressure and venous return. This sudden shift in preload and afterload can increase myocardial oxygen consumption and repolarization heterogeneity, triggering malignant arrhythmias or mechanical complications [6]; furthermore, chronic intestinal stasis promotes gut–heart axis dysfunction, characterized by gut barrier impairment and dysbiosis. This facilitates the translocation of gut‐derived inflammatory mediators—such as lipopolysaccharide (LPS) and trimethylamine N‐oxide (TMAO)—into the circulation, sustaining a prothrombotic and proinflammatory state that accelerates atherosclerosis and worsens long‐term cardiovascular prognosis [7, 8]. Consistent with these mechanisms, large‐scale evidence underscores this risk: A UK Biobank analysis of 400,000 participants linked constipation to a twofold increase in MACE risk [9], whereas the US VA cohort reported 10%–20% higher all‐cause and cardiovascular mortality [10]. Among post‐MI patients specifically, comorbid constipation is associated with a doubled risk of 6‐month heart failure readmission (HR = 2.12, 95% CI 1.07–4.19) [11].

Given the significant risks of constipation, the use of laxatives for prevention or management has become routine clinical practice for these patients. However, in the specific patient population of AMI, the choice of laxative is often based on personal experience rather than evidence‐based medicine, a practice fraught with potential risks [12, 13]. For instance, stimulant laxatives may increase myocardial oxygen demand by inducing intestinal cramping and pain; hypertonic preparations, such as sodium phosphate or magnesium‐based laxatives, can exacerbate hemodynamic instability by inducing significant intraluminal fluid shifts; and diarrhea from any laxative overuse may cause fatal electrolyte disturbances, triggering malignant arrhythmias in the setting of an ischemic myocardium [14, 15]. Therefore, identifying which laxative administration strategy is associated with more favorable cardiovascular outcomes and prognosis is a critical, unanswered clinical question.

Accordingly, this study is aimed at leveraging a large clinical database to compare the associations between three different types of initial laxative strategies and the cardiovascular and clinical outcomes of ICU patients with AMI.

## 2. Materials and Methods

### 2.1. Data Sources

This retrospective cohort study was conducted using data sourced from two accessible intensive care databases: the Medical Information Mart for Intensive Care IV (MIMIC‐IV) (v3.1). MIMIC‐IV contains comprehensive clinical data for over 450,000 hospitalizations at the Beth Israel Deaconess Medical Center from 2008 to 2019. The author, Bingfu Wang, was authorized to access both databases via the PhysioNet platform upon completion of the Collaborative Institutional Training Initiative (CITI) Program′s human subjects research course (Certificate No. 13971877).

**Figure 1:**
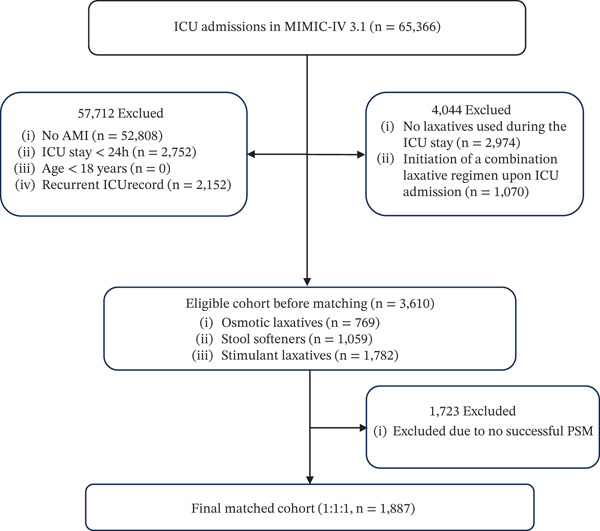
Study participant screening flowchart. Note: ICU, intensive care unit; MIMIC‐IV, Medical Information Mart for Intensive Care IV; AMI, acute myocardial infarction; PSM, propensity score matching.

This study enrolled patients with a first‐time hospitalization for AMI who were admitted to the ICU. Diagnostic classification was performed using the International Classification of Diseases (ICD). The ICD codes utilized to identify AMI included I2101, I2102, I2109, I2111, I2119, I2121, I2129, I213, I214, I219, I21A1, I21A9, I222, 41001, 41002, 41010, 41011, 41012, 41021, 41022, 41031, 41032, 41040, 41041, 41042, 41051, 41071, 41072, 41081, 41082, 41090, 41091, and 41092. Exclusion criteria were as follows: (1) ICU stay less than 24 h, (2) multiple ICU admissions due to AMI—only the first admission was included, and (3) patients who did not receive a single‐agent laxative as their initial therapy in the ICU (including those who received no laxatives or initial combination therapy). The inclusion and exclusion process is illustrated in the flowchart below (Figure 1).

### 2.2. Data Extraction

Patient data extraction during hospital admission was performed utilizing Navicat Premium (Version 16.1.15) via structured query language (SQL), encompassing demographic information (age, sex, and body mass index [BMI] [kg/m^2^]); comorbid conditions (hypertension, acute kidney injury, cerebrovascular accident, heart failure, chronic obstructive pulmonary disease, diabetes mellitus, hepatitis, and atrial fibrillation); pharmacotherapy (antiplatelet agents, *β*‐blockers, angiotensin‐converting enzyme inhibitors/angiotensin II receptor blockers, anticoagulants, statins, amiodarone, mineralocorticoid receptor antagonists, calcium channel blockers, and diuretics); vital signs (systolic blood pressure, diastolic blood pressure, heart rate, respiratory rate, and saturation of peripheral oxygen [SpO_2_]); laboratory parameters: hematology (hemoglobin, red blood cell distribution width, red blood cell count, white blood cell count, and absolute neutrophil count), percentage glycated hemoglobin, biochemistry (total calcium, chloride, globulin, glucose, potassium, total protein, sodium, total carbon dioxide, and lactate), blood gas analysis (partial pressure of carbon dioxide [pCO_2_], and potential of hydrogen [pH]), coagulation profile (D‐dimer, functional fibrinogen, international normalized ratio, prothrombin time, partial thromboplastin time, and thrombin time), lipid panel (high‐density lipoprotein cholesterol, low‐density lipoprotein cholesterol, total cholesterol, and triglycerides), hepatic function indices (direct bilirubin, indirect bilirubin, and total bilirubin), renal function markers (blood urea nitrogen and uric acid), myocardial injury biomarkers (creatine kinase MB isoenzyme, N‐terminal pro‐B‐type natriuretic peptide, and troponin T), urinalysis (urinary albumin, urinary albumin‐to‐creatinine ratio, bilirubin, leukocyte esterase, nitrite, and protein); and clinical severity scores: Sequential Organ Failure Assessment (SOFA) and Oxford Acute Severity of Illness Score (OASIS). Variables exhibiting missing values exceeding 30% were excluded from analysis, and those below this threshold were subjected to multiple imputation for missing data handling. Collected variables were taken from the earliest measurements after ICU admission.

### 2.3. Classification of Laxatives

To align with real‐world clinical practice, this study focuses on the effect of the initial laxative monotherapy post‐ICU admission in patients with AMI. This design isolates the independent impact of initial clinical decisions on prognosis. Subsequent medication changes or escalations due to insufficient efficacy are treated as downstream outcomes of the initial therapy rather than cohort crossovers. Patients initially receiving combined laxatives were excluded from the analysis. Based on the first monotherapy prescription post‐ICU admission, patients were stratified into three mutually exclusive cohorts according to the initial laxative agent administered following ICU admission. The osmotic laxative group comprised individuals initiated on oral osmotic agents, a broad category that includes nonabsorbable sugars (lactulose and sorbitol), polyethylene glycol (PEG), and saline agents. The latter primarily included magnesium‐containing products—such as magnesium hydroxide, magnesium citrate, magnesium oxide, and magnesium sulfate—as well as sodium phosphates. The stool softener group was defined by the exclusive first‐line use of docusate sodium, whereas the stimulant laxative group consisted of patients prescribed either senna or bisacodyl.

### 2.4. Outcome

The primary outcome was 28‐day all‐cause mortality following ICU admission. Secondary outcomes included in‐hospital mortality, ICU mortality, 365‐day mortality, and several ICU‐acquired complications: delirium, malignant arrhythmia, bowel sound recovery, and cardiogenic shock. The incidence of ICU‐acquired delirium was determined using the Confusion Assessment Method for the Intensive Care Unit (CAM‐ICU). A patient was considered to have developed delirium if they had at least one positive CAM‐ICU assessment during their ICU stay. Malignant arrhythmia was a composite endpoint that included the occurrence of any of the following during the ICU stay: persistent ventricular tachycardia, ventricular fibrillation, or cardiac arrest. Bowel sound recovery was defined as a transition from “absent” or “hypoactive” bowel sounds upon ICU admission to “present” or “hyperactive” before ICU discharge.

### 2.5. Statistical Analysis

Normality tests were initially conducted on continuous variables. Data conforming to a normal distribution were analyzed using the Student′s t‐test and expressed as mean ± standard deviation (SD). Non‐normally distributed variables were assessed via the Kruskal–Wallis test and presented as median with interquartile range (IQR). Categorical variables were evaluated using the Pearson′s chi‐squared test.

To control for baseline confounding, we performed 1:1:1 propensity score matching (PSM). Propensity scores were derived from a multinomial logistic regression model that included 34 baseline covariates, resulting in a final matched cohort of 629 patients per group. These covariates included age, sex, BMI, atrial fibrillation, diabetes mellitus, hypertension, hepatic disease, cerebrovascular accident, chronic kidney disease, heart failure, and chronic obstructive pulmonary disease; (initial laboratory values) white blood cell count, platelet count, hemoglobin, potassium, sodium, lactate, total bilirubin, creatinine, glucose, pH, SpO_2_, and systolic blood pressure; (disease severity scores) SOFA and OASIS; and (initial treatments) antiplatelet therapy, heparin, statins, beta‐blockers, diuretics, mineralocorticoid receptor antagonists, angiotensin‐converting enzyme inhibitors/angiotensin II receptor blockers, continuous renal replacement therapy, and mechanical ventilation. Intergroup balance in the matched cohort was confirmed by standardized mean differences below 0.1 (Table S1).

In the matched cohort, multivariate regression models were constructed to assess the independent effect of different laxative strategies on outcomes and to adjust for residual confounding. Conditional logistic regression, stratified by matched set ID (subclass), was used for binary outcomes, whereas a robust Cox proportional hazards model was employed for survival outcomes. The covariates for all models were selected using a two‐step process: Variables with a p value < 0.1 in univariate analysis were first identified, followed by a bidirectional stepwise regression based on the Akaike information criterion (AIC) to build the final parsimonious model. This procedure resulted in the inclusion of 15 key covariates for final adjustment: age; heart failure; chronic obstructive pulmonary disease; lactate, platelet count, total bilirubin, and glucose levels; SOFA and OASIS scores; and the use of diuretics, mineralocorticoid receptor antagonists, angiotensin‐converting enzyme inhibitors/angiotensin II receptor blockers, beta‐blockers, antiplatelet agents, and statins. The VIF values of the variables included in the analysis were all < 5 (Table S2). Furthermore, to address potential selection bias arising from the exclusion of unmatched patients in the PSM, a sensitivity analysis was conducted using inverse probability of treatment weighting (IPTW). We confirmed the balance of covariates through standardized mean differences (SMD < 0.1), then applied weighted regression models to the entire cohort (Table S3).

All statistical analyses were conducted using Python 3.10.6 and R software Version 4.3.2, with a two‐tailed p value of < 0.05 considered statistically significant.

## 3. Results

### 3.1. Baseline Characteristics

Before PSM, the three initial laxative treatment groups showed significant baseline differences (Table 1).

**Table 1:** Baseline data (MIMIC cohort).

	Stimulant laxatives (*n* = 1782)	Stool softeners (*n* = 1059)	Osmotic laxatives (n = 769)	*p* value
**Demographics**				
Age, years	72.00 (62.00, 81.00)	70.00 (63.00–78.00)	71.00 (62.00, 78.00)	< 0.001
Gender (male), n (%)	1112.00 (62.40%)	747.00 (70.54%)	529.00 (68.79%)	< 0.001
BMI, kg/m^2^	27.80 (24.25, 32.16)	28.62 (25.60, 32.91)	28.93 (25.55, 32.61)	< 0.001
**Comorbidities**				
CS, n (%)	343.00 (19.25%)	104.00 (9.82%)	95.00 (12.35%)	< 0.001
MA, n (%)	250.00 (14.03%)	91.00 (8.59%)	75.00 (9.75%)	< 0.001
CVA, n (%)	162.00 (9.09%)	105.00 (9.92%)	63.00 (8.19%)	0.449
Delirium, n (%)	477.00 (26.77%)	176.00 (16.62%)	193.00 (25.10%)	< 0.001
AF, n (%)	454.00 (25.48%)	247.00 (23.32%)	169.00 (21.98%)	0.129
HTN, n (%)	647.00 (36.31%)	501.00 (47.31%)	318.00 (41.35%)	< 0.001
HEP, n (%)	66.00 (3.70%)	36.00 (3.40%)	78.00 (10.14%)	< 0.001
HF, n (%)	896.00 (50.28%)	417.00 (39.38%)	350.00 (45.51%)	< 0.001
COPD, n (%)	278.00 (15.60%)	161.00 (15.20%)	116.00 (15.08%)	0.931
DM, n (%)	742.00 (41.64%)	507.00 (47.88%)	352.00 (45.77%)	0.004
**Medication**				
Antiplatelet, n (%)	1550.00 (86.98%)	984.00 (92.92%)	632.00 (82.18%)	< 0.001
Beta‐blockers, n (%)	1426.00 (80.02%)	966.00 (91.22%)	637.00 (82.83%)	< 0.001
ACEI/ARB, n (%)	775.00 (43.49%)	337.00 (31.82%)	224.00 (29.13%)	< 0.001
Heparin, n (%)	1590.00 (89.23%)	698.00 (65.91%)	549.00 (71.39%)	< 0.001
Statins, n (%)	1368.00 (76.77%)	904.00 (85.36%)	617.00 (80.23%)	< 0.001
Amiodarone, n (%)	339.00 (19.02%)	267.00 (25.21%)	204.00 (26.53%)	< 0.001
MRA, n (%)	90.00 (5.05%)	16.00 (1.51%)	30.00 (3.90%)	< 0.001
CCB, n (%)	295.00 (16.55%)	202.00 (19.07%)	117.00 (15.21%)	0.074
Diuretics, n (%)	376.00 (21.10%)	307.00 (28.99%)	217.00 (28.22%)	< 0.001
**Vital signs**				
SBP, mmHg	119.00 (106.00, 136.00)	114.00 (100.00, 128.00)	113.00 (100.00, 127.00)	< 0.001
DBP, mmHg	68.00 (58.00, 80.00)	62.00 (53.00, 71.00)	62.00 (54.00, 72.00)	< 0.001
HR, beats/min	84.00 (73.00, 98.00)	80.00 (74.00, 88.00)	84.00 (76.00, 94.00)	< 0.001
RR, breaths/min	19.00 (16.00, 23.00)	16.00 (13.00, 18.00)	16.00 (13.00, 21.00)	< 0.001
SpO_2_, %	98.00 (95.00, 100.00)	100.00 (97.00, 100.00)	99.00 (96.00, 100.00)	< 0.001
**Lab data**				
Hemoglobin, g/dL	11.00 (9.30, 12.70)	9.60 (8.30, 11.30)	9.40 (8.20, 10.90)	< 0.001
WBC, ×10^9^/L	11.80 (8.70, 15.80)	12.40 (9.10, 16.20)	12.10 (8.80, 16.20)	0.021
PLT, ×10^9^/L	208.00 (157.00, 265.00)	159.00 (124.00, 209.00)	171.00 (130.00, 226.00)	< 0.001
Cl^−^, mmol/L	103.00 (99.00, 106.00)	103.00 (99.00, 106.00)	101.50 (97.00, 106.00)	0.018
K^+^, mmol/L	4.30 (3.90, 4.70)	4.30 (4.00, 4.70)	4.30 (4.00, 4.80)	0.043
Na^+^, mmol/L	138.00 (135.00, 141.00)	138.00 (136.00, 140.00)	138.00 (136.00, 140.00)	0.894
Lactate, mmol/L	1.70 (1.20, 2.60)	1.90 (1.40, 2.60)	2.00 (1.40, 2.90)	< 0.001
pCO_2_, mmHg	41.00 (36.00, 47.00)	40.00 (36.00, 44.00)	40.00 (36.00, 45.00)	< 0.001
pH	7.37 (7.30, 7.41)	7.40 (7.35, 7.43)	7.39 (7.34, 7.44)	< 0.001
TBIL, mg/dL	0.60 (0.40, 0.90)	0.60 (0.40, 0.90)	0.60 (0.40, 1.10)	< 0.001
Glucose, mg/dL	138.00 (112.00, 190.00)	125.00 (107.00, 152.00)	124.00 (105.00, 155.00)	< 0.001
ALT, U/L	33.00 (19.00, 78.00)	26.00 (16.00, 53.00)	27.00 (17.00, 59.00)	< 0.001
Cr, mg/dL	1.20 (0.90, 1.80)	1.00 (0.80, 1.30)	1.00 (0.80, 1.70)	< 0.001
BUN, mmol/L	23.00 (15.00, 39.00)	18.00 (14.00, 26.00)	20.00 (14.00, 36.00)	< 0.001
TnT, ng/mL	0.67 (0.18, 2.18)	0.52 (0.19, 1.37)	0.41 (0.14, 1.09)	< 0.001
**Organ support**				
CRRT	114.00 (6.40%)	58.00 (5.48%)	86.00 (11.18%)	< 0.001
Mechanical ventilation	1,514.00 (84.96%)	1,009.00 (95.28%)	729.00 (94.80%)	< 0.001
**Clinical scoring**				
OASIS	31.00 (26.00, 38.00)	32.00 (27.00, 38.00)	33.00 (28.00, 40.00)	< 0.001
SOFA	4.00 (2.00, 7.00)	5.00 (3.00, 7.00)	6.00 (4.00, 9.00)	< 0.001

Note: Continuous variables were presented as medians (IQR) and compared using the Kruskal–Wallis test. Categorical variables were presented as frequencies (%) and compared using the Pearson′s chi‐squared test. Comparisons were made across the three initial laxative strategy groups.

Abbreviations: ACEI/ARB, angiotensin‐converting enzyme inhibitor/angiotensin II receptor blocker; AF, atrial fibrillation; ALT, alanine aminotransferase; BMI, body mass index; BUN, blood urea nitrogen; CCB, calcium channel blocker; Cl^−^, chloride; COPD, chronic obstructive pulmonary disease; Cr, creatinine; CRRT, continuous renal replacement therapy; CS, cardiogenic shock; CVA, cerebrovascular accident; DBP, diastolic blood pressure; DM, diabetes mellitus; HEP, hepatic disease; HF, heart failure; HR, heart rate; HTN, hypertension; K^+^, potassium; MA, malignant arrhythmia; MRA, mineralocorticoid receptor antagonist; Na^+^, sodium; OASIS, Oxford Acute Severity of Illness Score; pCO_2_, partial pressure of carbon dioxide; PLT, platelet count; pH, potential of hydrogen; RR, respiratory rate; SBP, systolic blood pressure; SOFA, Sequential Organ Failure Assessment; SpO_2_, saturation of peripheral oxygen; TBIL, total bilirubin; TnT, troponin T; WBC, white blood cell count.

The stimulant laxative group (n = 1782) was the oldest (median 72 years, p < 0.001) and had the highest incidence of in‐ICU cardiogenic shock (19.25%) and malignant arrhythmia (14.03%) (both p < 0.001). Initial vital signs also pointed to instability, with this group having the highest systolic blood pressure and respiratory rate (both p < 0.001). Despite this, their disease severity scores at ICU admission (median SOFA 4.0, median OASIS 31.0) were significantly lower than in the other two cohorts (both p < 0.001).

In contrast, patients initiated on stool softeners (n = 1059) and osmotic laxatives (n = 769) presented with significantly higher SOFA and OASIS scores (both p < 0.001) and required mechanical ventilation more frequently (95.3% and 94.8%, respectively, p < 0.001). Medication patterns during the ICU stay also varied markedly. Use of antiplatelet agents, beta‐blockers, and lipid‐lowering agents was most frequent in the stool softener group. Conversely, heparin and renin–angiotensin system inhibitors were most commonly administered to patients receiving stimulant laxatives. Meanwhile, amiodarone and diuretics were used more often in the stool softener and osmotic laxative groups than in the stimulant group (all p < 0.001).

These extensive baseline differences across demographics, in‐ICU complications, vital signs, disease severity scores, and in‐ICU medication patterns confirm significant treatment selection bias in clinical practice, underscoring the need for PSM.

### 3.2. Research Findings

#### 3.2.1. Clinical Outcomes of Patient Cohorts

In the initial cohort before PSM, clinical outcomes differed markedly among treatment groups (Table 1). In the stimulant, stool softener, and osmotic laxative groups, ICU mortality was 11.45%, 6.14%, and 12.09%, respectively; in‐hospital mortality was 16.44%, 8.22%, and 14.82%; 28‐day mortality was 19.42%, 8.69%, and 16.12%; and 365‐day mortality was 33.95%, 16.43%, and 28.22%. For in‐ICU complications, the incidence of cardiogenic shock was 19.25%, 9.82%, and 12.35%; malignant arrhythmia was 14.03%, 8.59%, and 9.75%; and delirium was 26.77%, 16.62%, and 25.10%, respectively. The rate of bowel sound recovery was 18.07%, 47.40%, and 42.91% across the three groups.

After 1:1:1 PSM in a cohort of 1887 patients (n = 629 per group), the distribution of outcomes was reassessed. In the stimulant, stool softener, and osmotic laxative groups, ICU mortality was 12.88%, 9.38%, and 12.88%, respectively. In‐hospital mortality was 17.65%, 12.08%, and 15.26% in the respective groups. The 28‐day mortality rates were 19.71%, 12.24%, and 17.17%, and 365‐day mortality rates were 32.43%, 21.14%, and 29.57%. Regarding in‐ICU complications, cardiogenic shock occurred in 17.01%, 13.67%, and 13.35% of patients; malignant arrhythmia in 11.13%, 11.76%, and 10.81%; and delirium in 29.89%, 19.40%, and 25.60%, respectively. Finally, bowel sound recovery was achieved in 28.30%, 41.65%, and 38.95% of patients in the respective groups.

#### 3.2.2. Kaplan–Meier Curves for 28‐Day and 365‐Day Mortality by Laxative Strategy

Kaplan–Meier survival analysis (Figure 2) revealed significant differences in survival curves among the three groups at both 28 days (log − rank p = 0.0015) and 365 days (log − rank p < 0.0001). In the short term, a clear hierarchy of survival benefit was observed: The stool softener group had the highest survival, followed by the osmotic group, and lastly the stimulant group. This pattern evolved during long‐term follow‐up: The survival advantage of the stool softener group became more pronounced, with its curve remaining significantly above the other two. Meanwhile, the survival curves for the osmotic and stimulant groups, while remaining close to each other, were both substantially lower than that of the stool softener group.

Figure 2Kaplan–Meier curves for 28‐day and 365‐day mortality by laxative strategies. Note: Survival analysis for 28‐day (a) and 365‐day (b) all‐cause mortality in the propensity score‐matched cohort (n = 629 per group), categorized by the initial laxative strategy administered: stimulant, stool softener, or osmotic. p values derived from the log‐rank test are displayed on each plot.

**Figure:**
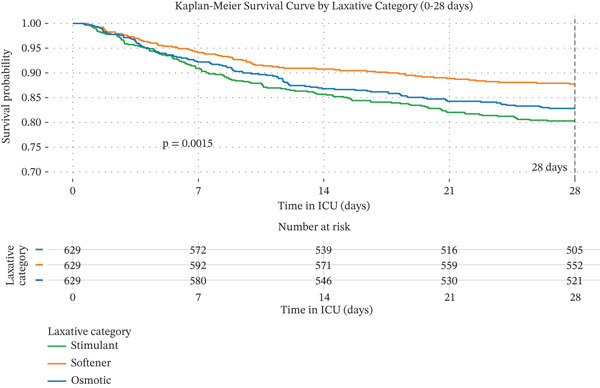
(a)

**Figure:**
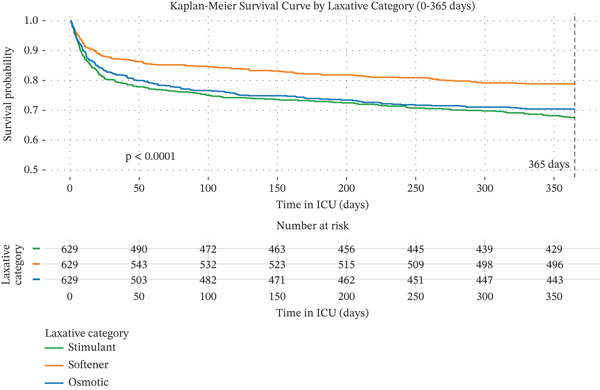
(b)

#### 3.2.3. In‐Hospital and ICU Mortality

**Figure 3:**
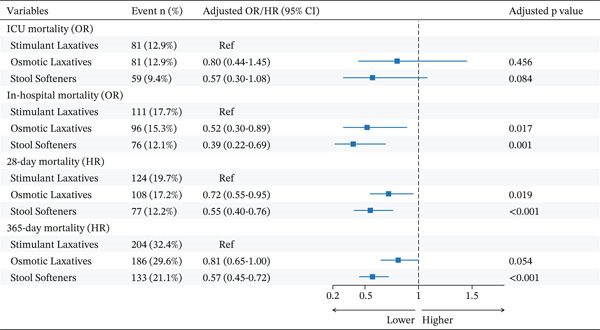
Forest plot for adjusted mortality outcomes. Note: Forest plot for the adjusted analysis of short‐term (ICU and in‐hospital) and long‐term (28‐ and 365‐day) mortality. Odds ratios (ORs) are reported for ICU and in‐hospital mortality, whereas hazard ratios (HRs) are reported for 28‐day and 365‐day mortality. Both effect sizes compare osmotic laxatives and stool softeners to the stimulant laxatives reference group. Abbreviations: CI, confidence interval; HR, hazard ratio; ICU, intensive care unit; OR, odds ratio; Ref, reference.

In the PSM cohort, we assessed the association between initial laxative strategies and in‐hospital and ICU mortality (Figure 3 and Table S4). Although the use of stool softeners was associated with lower ICU mortality in the unadjusted model compared with stimulant laxatives (OR = 0.65, 95% CI 0.44–0.97, p = 0.033), this association did not reach statistical significance after full adjustment (p = 0.084). For in‐hospital mortality, however, significant associations were observed in the fully adjusted model, with both stool softeners (OR = 0.39, 95% CI 0.22–0.69, p = 0.001) and osmotic laxatives (OR = 0.52, 95% CI 0.30–0.89, p = 0.017) being linked to a lower risk of death compared with stimulant laxatives.

To assess the robustness of these findings, a sensitivity analysis using IPTW was performed (Table S6). The results were largely consistent with the primary analysis. For in‐hospital mortality, IPTW similarly demonstrated that both stool softeners (p < 0.001) and osmotic laxatives (p < 0.001) were significantly associated with a lower risk of death in the adjusted IPTW model. The main difference emerged in the analysis of ICU mortality; in the IPTW model, the use of stool softeners was significantly associated with lower ICU mortality (p = 0.002) in the adjusted IPTW model, an association that was not statistically significant in the adjusted PSM model. The p for trend for in‐hospital mortality was statistically significant in both analyses (p ≤ 0.003).

#### 3.2.4. 16‐ and 365‐Day Mortality

In the PSM cohort, we assessed the association between initial laxative strategies and 28‐day and 365‐day mortality (Figure 3 and Table S4). The use of stool softeners was consistently associated with a significantly lower risk of both 28‐day mortality (adjusted HR = 0.55, 95% CI 0.40–0.76, p < 0.001) and 365‐day mortality (adjusted HR = 0.57, 95% CI 0.45–0.72, p < 0.001) compared with stimulant laxatives. For osmotic laxatives, an association with lower 28‐day mortality was observed after multivariate adjustment (HR = 0.72, 95% CI 0.55–0.95, p = 0.019); however, this link did not reach statistical significance for 365‐day mortality (p = 0.054).

To assess the robustness of these findings, a sensitivity analysis using IPTW was performed (Table S6). The results of this analysis were largely consistent with the primary findings, again demonstrating that stool softener use was significantly associated with lower 28‐day (p < 0.001) and 365‐day mortality (p < 0.001) compared with stimulant laxatives in the adjusted IPTW model. The primary distinction from the main analysis was that the association between osmotic laxatives and 28‐day mortality did not reach statistical significance in the adjusted IPTW model (p = 0.097). Notably, a significant linear trend was observed across both analyses for 28 and 365‐day mortality outcomes (all p for trend < 0.001).

#### 3.2.5. Cardiogenic Shock and Malignant Arrhythmia

**Figure 4:**
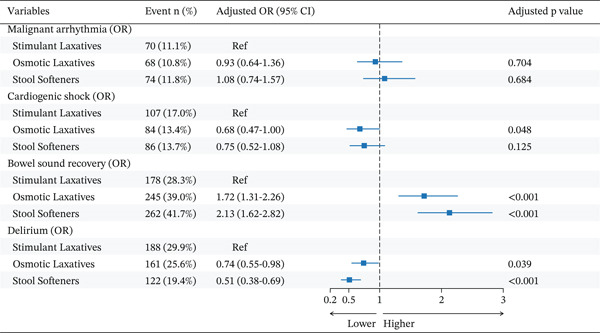
Forest plot for clinical complications and recovery outcomes. Note: Forest plot for the adjusted analysis of clinical complications (malignant arrhythmia, cardiogenic shock, and delirium) and recovery indicators (bowel sound recovery). Odds ratios (ORs) with 95% confidence intervals (CIs) compare osmotic laxatives and stool softeners to the stimulant laxatives reference group. For clinical complications, an OR < 1 indicates a lower risk; for bowel sound recovery, an OR > 1 indicates a higher likelihood of recovery. Abbreviations: CI, confidence interval; OR, odds ratio; Ref, reference.

For malignant arrhythmia, the two analytical methods yielded conflicting results. In the PSM analysis (Figure 4 and Table S5), no significant association with risk was observed for either stool softeners or osmotic laxatives in both unadjusted and adjusted models compared with stimulant laxatives (all p > 0.05). In stark contrast, the IPTW sensitivity analysis (Table S7) revealed that the use of both stool softeners (adjusted p = 0.006) and osmotic laxatives (adjusted p = 0.004) was significantly associated with a lower risk of malignant arrhythmia.

Regarding cardiogenic shock, the PSM analysis (Figure 4 and Table S5) showed a limited association, with only osmotic laxatives being linked to a lower risk after multivariate adjustment (OR = 0.68, 95% CI 0.47–1.00, p = 0.048). By contrast, the IPTW analysis (Table S7) revealed a stronger and more consistent protective association. In this model, both stool softeners (p < 0.001) and osmotic laxatives (p < 0.001) were significantly associated with a lower risk of cardiogenic shock, an association that remained robust after multivariate adjustment (p < 0.001).

#### 3.2.6. Bowel Sound Recovery and Delirium

In the assessment of functional outcomes and delirium, significant associations were observed for different laxative strategies (Figure 4 and Table S5). For bowel sound recovery, both stool softeners (adjusted OR = 2.13, 95% CI 1.62–2.82, p < 0.001) and osmotic laxatives (adjusted OR = 1.72, 95% CI 1.31–2.26, p < 0.001) were significantly associated with a higher likelihood of recovery compared with stimulant laxatives in the matched cohort. Regarding delirium, a protective association was noted, with both stool softeners (adjusted OR = 0.51, 95% CI 0.38–0.69, p < 0.001) and osmotic laxatives (adjusted OR = 0.74, 95% CI 0.55–0.98, p = 0.039) being linked to a significantly lower risk.

The sensitivity analysis using IPTW strongly corroborated these findings (Table S7). This analysis likewise demonstrated that both stool softeners (p < 0.001) and osmotic laxatives (p < 0.001) were significantly associated with a higher likelihood of bowel sound recovery and a lower risk of delirium (p < 0.001 and p = 0.001, respectively) compared with stimulant laxatives after multivariate adjustment. The high degree of consistency between the two analytical approaches reinforces the robustness of these results.

#### 3.2.7. Subgroup Analyses

**Figure 5:**
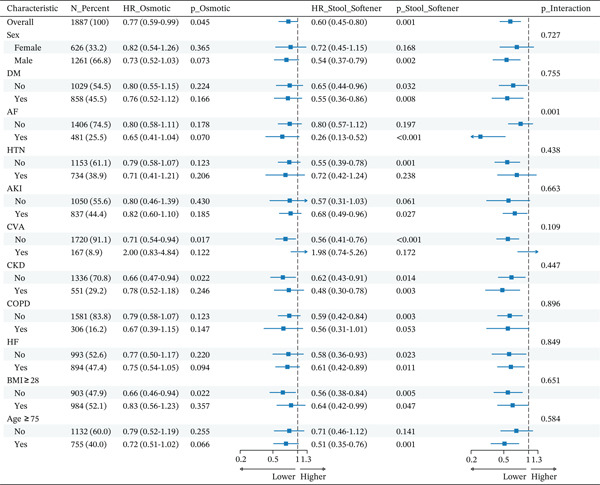
Forest plot for subgroup analyses of 28‐day mortality. Note: Forest plot for the subgroup analysis of 28‐day all‐cause mortality in the propensity score–matched cohort. Hazard ratios (HRs) compare osmotic and stool softener laxatives with the stimulant reference group. The p for interaction assesses for consistency of effect across subgroups. Abbreviations: AF, atrial fibrillation; AKI, acute kidney injury; BMI, body mass index; CKD, chronic kidney disease; COPD, chronic obstructive pulmonary disease; CVA, cerebrovascular accident; DM, diabetes mellitus; HF, heart failure; HTN, hypertension.

To assess the consistency of the associations between laxative strategies and 28‐day mortality, we performed subgroup analyses (Figure 5), using the stimulant laxative group as the reference. Tests for interaction revealed that the protective effects of stool softeners and osmotic laxatives were robust across most prespecified subgroups. A significant interaction was observed for the atrial fibrillation subgroup (p for interaction = 0.001). Stool softeners exhibited a stronger protective effect in patients with atrial fibrillation (HR = 0.26, 95% CI 0.13–0.52) compared with those without atrial fibrillation (HR = 0.55, 95% CI 0.36–0.86), suggesting that the survival benefit may be magnified in this specific population.

## 4. Discussion

Our study revealed that the initial choice of laxative strategy was significantly associated with both short‐ and long‐term mortality in patients admitted to the ICU for AMI. In the primary PSM analysis, stool softeners were linked to significantly lower in‐hospital mortality (adjusted OR = 0.39; 95% CI, 0.22–0.69; p = 0.001) and 28‐day mortality (adjusted HR = 0.55; 95% CI, 0.40–0.76; p < 0.001) when compared with stimulant laxatives. A similar protective association was observed for osmotic laxatives, which were also linked to lower rates of in‐hospital mortality (adjusted OR = 0.52; 95% CI, 0.30–0.89; p = 0.017) and 28‐day mortality (adjusted HR = 0.72; 95% CI, 0.55–0.95; p = 0.019). Notably, this protective effect for stool softeners remained robust at the 365‐day follow‐up (adjusted OR = 0.57; 95% CI, 0.45–0.72; p < 0.001), whereas no such sustained benefit was observed for osmotic laxatives (p = 0.054). These core findings were reinforced by the IPTW sensitivity analysis.

In our prespecified subgroup analysis, a significant effect modification was observed for atrial fibrillation (p for interaction = 0.001), with stool softeners exhibiting a more pronounced association with survival in this population. Theoretically, AF patients are more vulnerable to the autonomic fluctuations and hemodynamic instability induced by the Valsalva maneuver during defecation [17, 18]; stool softeners may mitigate these triggers by reducing straining. Furthermore, stool softeners are less likely to cause diarrhea and subsequent electrolyte imbalances, which are known to exacerbate AF burden [19, 20]. However, as AF patients comprised only 25% of our cohort, these subgroup findings should be interpreted with caution due to potential multiple testing bias and residual confounding.

Stool softeners soften feces by lowering surface tension rather than stimulating enteric nerves or forcing peristalsis. Theoretically, this mild mechanism mitigates excessive straining and the Valsalva maneuver, potentially averting dangerous hemodynamic fluctuations and minimizing acute cardiovascular stress without disrupting fluid balance or triggering visceral reflexes [21]. In contrast, the invasive mechanism of stimulant laxatives theoretically poses a dual threat: Intense enteric nerve stimulation might increase myocardial oxygen demand via visceral–cardiac reflexes, whereas direct mucosal irritation could compromise the intestinal barrier and increase permeability [22, 23]. Clinically, AMI patients frequently exhibit elevated serum LPS and gut barrier biomarkers, which correlate with adverse long‐term outcomes [24]. Concurrently, basic research demonstrates that barrier‐mediated LPS translocation activates systemic inflammatory and coagulation pathways (e.g., TLR4), driving atherosclerotic plaque instability, and thrombosis [25]. Consequently, this barrier‐inflammatory cascade, potentially triggered by stimulant laxatives, offers a plausible mechanistic hypothesis for their inferior cardiovascular safety profile.

The intermediate risk profile of osmotic laxatives observed in our cohort is hypothesized to stem from their mechanism, which might act as a “double‐edged sword” in AMI patients. On one hand, their core mechanism of drawing water into the intestinal lumen could theoretically serve as a beneficial “luminal diuresis” for patients with comorbid heart failure and volume overload, whereas magnesium absorption has been suggested to offer antiarrhythmic properties [26]. On the other hand, this fluid shift poses a theoretical risk of exacerbating deficits in effective circulating volume among hemodynamically unstable patients. Furthermore, previous studies have shown that oral magnesium and phosphate salts can be associated with hypermagnesemia and acute kidney injury in those with renal impairment [27, 28].

A core finding of our study is that, compared with stimulant laxatives, stool softeners are associated with the lowest short‐ and long‐term mortality, demonstrating a superior safety profile. However, lacking robust efficacy data, the 2023 AGA/ACG constipation guidelines excluded docusate from recommended treatments [16], prompting widespread institutional de‐implementation of this low‐value medication[29, 30]. Therefore, we hypothesize that for AMI patients, the primary benefit of stool softeners lies in reducing defecation burden rather than resolving true constipation. This prophylactic strategy—preventing the Valsalva maneuver and its hemodynamic stress—could theoretically explain the favorable safety profile observed among stool softener users in our cohort, potentially underscoring its unique clinical value in this specific population.

However, when stool softeners fail to alleviate refractory constipation, osmotic laxatives—particularly PEG—might represent an optimal clinical step‐up option. Previous research demonstrates PEG′s superior efficacy and safety over other osmotic agents [31, 32]. Lactulose, a nonabsorbable oligosaccharide, undergoes fermentation by colonic bacteria, frequently causing flatulence, abdominal pain, and excessive passing of wind; meanwhile, magnesium salts and phosphates carry distinct risks of electrolyte imbalance or renal dysfunction (e.g., magnesium retention and phosphate nephropathy) [33, 34]. We therefore hypothesize that in specific clinical scenarios—such as overt constipation or ineffective initial stool softeners in AMI patients—PEG could offer a superior risk‐benefit ratio, warranting future prospective confirmation.

This study has several key strengths. First, it includes a large sample of 3610 AMI patients from the MIMIC‐IV database. Second, its doubly robust methodology, using both PSM and IPTW, minimizes baseline confounding, thus strengthening the validity of our findings. Third, we assessed a wide range of outcomes, from hard endpoints like mortality to key ICU complications such as cardiogenic shock and malignant arrhythmias. Finally, it addresses a common but evidence‐poor clinical question, providing crucial evidence to guide the choice of laxatives for AMI patients. However, the study has inherent limitations. First, its retrospective, observational design can only establish association, not causation. Second, the single‐region, US‐based data may limit the generalizability of our results. Lastly, database limitations precluded the assessment of direct efficacy endpoints, such as bowel frequency and stool consistency. Future granular prospective studies are required to collect objective data and comprehensively ascertain the exact risk‐benefit profiles of various laxatives, with a particular focus on evaluating promising agents like PEG. Furthermore, to better guide clinical practice, these future trials must explicitly stratify clinical scenarios into “routine prophylaxis” versus “treating existing constipation” in AMI patients.

## 5. Conclusion

Among ICU‐admitted AMI patients, our observational data suggest that the initial use of stool softeners or osmotic laxatives was associated with a lower risk of short‐term mortality compared with stimulant laxatives. These findings indicate that a more robust and gentle laxative management strategy may have potential clinical value for this high‐risk population.

## Funding

This study was supported by the National Natural Science Foundation of China (10.13039/501100001809) (No. 81202852), the Tianjin Jinmen Medical Talents Project (No. TISJMYXYC‐D2‐052), and the Tianjin Chinese Medicine Key Scientific Research Project (No. 2024003).

## Ethics Statement

This study utilized data from the Medical Information Mart for Intensive Care IV (MIMIC‐IV, v2.0). The use of MIMIC‐IV was approved by the Institutional Review Boards of Beth Israel Deaconess Medical Center (IRB No. 2001‐P‐001699/14) and the Massachusetts Institute of Technology (IRB No. 0403000206). As the study used only deidentified data, it was granted an exemption from further ethical approval by the Institutional Review Board of the First Teaching Hospital of Tianjin University of Traditional Chinese Medicine.

## Conflicts of Interest

The authors declare no conflicts of interest.

## Supporting information


**Supporting Information** Additional supporting information can be found online in the Supporting Information section. Table S1 Balance of baseline covariates before and after propensity score matching (PSM). Table S2: Variable selection process for the multivariable Cox regression model of 28‐day mortality, including univariate analysis, final multivariable model selection, and variance inflation factor (VIF). Table S3: Balance of baseline covariates before and after inverse probability of treatment weighting (IPTW). Table S4: Association of initial laxative strategy with short‐ and long‐term mortality (ICU, in‐hospital, 28‐day, and 365‐day) after PSM. Table S5: Association of initial laxative strategy with secondary clinical outcomes (malignant arrhythmia, cardiogenic shock, bowel sound recovery, and delirium) after PSM. Table S6: Association of initial laxative strategy with short‐ and long‐term mortality after IPTW. Table S7: Association of initial laxative strategy with secondary clinical outcomes after IPTW.

## Data Availability

Research data may be obtained upon reasonable request from the corresponding author.
