# Correction to “Association of Initial Laxative Strategy With Clinical Outcomes in Critically Ill Patients With Acute Myocardial Infarction: A Retrospective Cohort Study”

**DOI:** 10.1155/cdr/9891810

**Published:** 2026-06-24

B. Wang, Y. Song, Y. Fan, and Z. Zhao, “Association of Initial Laxative Strategy With Clinical Outcomes in Critically Ill Patients With Acute Myocardial Infarction: A Retrospective Cohort Study,” *Cardiovascular Therapeutics*, no. 2026 (2026). https://doi.org/10.1155/cdr/1649836.

In the article titled “Association of Initial Laxative Strategy With Clinical Outcomes in Critically Ill Patients With Acute Myocardial Infarction: A Retrospective Cohort Study,” there were multiple errors. These errors are shown and corrected below:

**Error in Section 2.1:**

The number of intensive care databases used in the study described was incorrect. The study only utilized the MIMIC‐IV dataset, and the relevant information in this section should be corrected as follows:

[“This retrospective cohort study was conducted using data sourced from two accessible intensive care databases: the Medical Information Mart for Intensive Care IV (MIMIC‐IV) (v3.1). MIMIC‐IV contains comprehensive clinical data for over 450,000 hospitalizations at the Beth Israel Deaconess Medical Center from 2008 to 2019. The author, Bingfu Wang, was authorized to access both databases via the PhysioNet platform upon completion of the Collaborative Institutional Training Initiative (CITI) Program′s human subjects research course (Certificate No. 13971877).”]

should read:

[“This retrospective cohort study was conducted using data sourced from the Medical Information Mart for Intensive Care IV (MIMIC‐IV) (v3.1) database. MIMIC‐IV contains comprehensive clinical data for over 450,000 hospitalizations at the Beth Israel Deaconess Medical Center from 2008 to 2019. The author, Bingfu Wang, was authorized to access the database via the PhysioNet platform upon completion of the Collaborative Institutional Training Initiative (CITI) Program′s human subjects research course (Certificate No. 13971877).”]

**Error in author affiliations:**

The affiliations of the listed authors Yulong Song, Yujian Fan, and Zhiqiang Zhao were incorrect. The corrected affiliations appear below:

Bingfu Wang^1,2,3^, Yulong Song^2,3^, Yujian Fan^2,3^, and Zhiqiang Zhao^2,3^

^1^Department of Cardiovascular Medicine, The First Affiliated Hospital of Henan University of Chinese Medicine, Zhengzhou, China

^2^First Teaching Hospital of Tianjin University of Traditional Chinese Medicine, Tianjin, China

^3^National Clinical Research Center for Chinese Medicine, Tianjin, China

We apologize for these errors.

