# Supplementary material for: Association of Initial Laxative Strategy With Clinical Outcomes in Critically Ill Patients With Acute Myocardial Infarction: A Retrospective Cohort Study
Source: Cardiovasc Ther. 2026 Apr 11;2026:1649836. doi: 10.1155/cdr/1649836 (PMC13069469; doi:10.1155/cdr/1649836)
Supplement: Supplementary file 1 — Supporting Information Additional supporting information can be found online in the Supporting Information section. Table S1 Balance of baseline covariates before and after propensity score matching (PSM). Table S2: Variable selection process for the multivariable Cox regression model of 28‐day mortality, including univariate analysis, final multivariable model selection, and variance inflation factor (VIF). Table S3: Balance of baseline covariates before and after inverse probability of treatment weighting (IPTW). Table S4: Association of initial laxative strategy with short‐ and long‐term mortality (ICU, in‐hospital, 28‐day, and 365‐day) after PSM. Table S5: Association of initial laxative strategy with secondary clinical outcomes (malignant arrhythmia, cardiogenic shock, bowel sound recovery, and delirium) after PSM. Table S6: Association of initial laxative strategy with short‐ and long‐term mortality after IPTW. Table S7: Association of initial laxative strategy with secondary clinical outcomes after IPTW. [file CDR-2026-1649836-s001.docx]

Supplementary Table S1. Balance of Baseline Covariates Before and After Propensity Score Matching (PSM)

| **Characteristic** | **Before PSM**  **SMD** | | **After PSM**  **SMD** |  |
| --- | --- | --- | --- | --- |
| **Demographics** |  |  | | |
| Age | 0.104 | | 0.033 |  |
| Gender (Male) | 0.081 | | 0.008 |  |
| BMI | 0.117 | | 0.038 |  |
| **Comorbidities** |  | |  |  |
| CVA | 0.017 | | 0.011 |  |
| AF | 0.035 | | 0.059 |  |
| HTN | 0.110 | | 0.029 |  |
| HEP | 0.034 | | 0.014 |  |
| HF | 0.109 | | 0.027 |  |
| COPD | 0.005 | | 0.018 |  |
| DM | 0.062 | | 0.022 |  |
| CKD | 0.040 | | 0.027 |  |
| **Medication** |  | |  |  |
| Anti-platelet | 0.107 | | 0.033 |  |
| Beta blockers | 0.112 | | 0.035 |  |
| ACEI/ARB | 0.144 | | 0.051 |  |
| Heparin | 0.233 | | 0.016 |  |
| Statins | 0.086 | | 0.018 |  |
| MRA | 0.035 | | 0.021 |  |
| Diuretics | 0.079 | | 0.005 |  |
| **Vital signs** |  | |  |  |
| SBP | 0.275 | | 0.064 |  |
| SpO₂ | 0.447 | | 0.059 |  |
| **Lab data** |  | |  |  |
| Hemoglobin | 0.585 | | 0.046 |  |
| WBC | 0.025 | | 0.034 |  |
| PLT | 0.474 | | 0.017 |  |
| K⁺ | 0.037 | | 0.051 |  |
| Na⁺ | 0.020 | | 0.022 |  |
| Lactate | 0.157 | | 0.013 |  |
| pH | 0.279 | | 0.079 |  |
| TBIL | 0.290 | | 0.048 |  |
| Glucose | 0.293 | | 0.017 |  |
| Cr | 0.172 | | 0.093 |  |
| **Organ support** |  | |  |  |
| CRRT | 0.057 | | 0.029 |  |
| Mechanical ventilation | 0.103 | | 0.022 |  |
| **Clinical scoring** |  | |  |  |
| OASIS | 0.173 | | 0.039 |  |
| SOFA | 0.417 | | 0.074 |  |

BMI, Body Mass Index; CVA, Cerebrovascular Accident; AF, Atrial Fibrillation; HTN, Hypertension; HEP, hepatic disease; HF, Heart Failure; COPD, Chronic Obstructive Pulmonary Disease; DM, Diabetes Mellitus; CKD, Chronic Kidney Disease; ACEI/ARB, Angiotensin-Converting Enzyme Inhibitor/Angiotensin II Receptor Blocker; MRA, Mineralocorticoid Receptor Antagonist; SBP, Systolic Blood Pressure; SpO₂, Saturation of Peripheral Oxygen; WBC, White Blood Cell Count; PLT, Platelet Count; K⁺, Potassium; Na⁺, Sodium; TBIL, Total Bilirubin; Cr, Creatinine; CRRT, Continuous Renal Replacement Therapy; OASIS, Oxford Acute Severity of Illness Score; SOFA, Sequential Organ Failure Assessment.

Supplementary Table S2. Variable Selection Process for the Multivariable Cox Regression Model of 28-Day Mortality

| **Variable** | **Univariate Analysis** | | **Multivariable Model Final Selection** | | **VIF** |  |
| --- | --- | --- | --- | --- | --- | --- |
| Initial Laxative Strategy | < 0.001 | ✓ | | 1.01 | | |
| **Demographics** |  | |  | |  |  |
| Age | < 0.001 | | ✓ | | 1.11 |  |
| Gender | 0.089 | |  | |  |  |
| BMI | 0.231 | |  | |  |  |
| **Comorbidities** |  | |  | |  |  |
| CVA | 0.058 | |  | |  |  |
| AF | 0.093 | |  | |  |  |
| HTN | 0.054 | |  | |  |  |
| HEP | 0.076 | |  | |  |  |
| HF | < 0.001 | | ✓ | | 1.14 |  |
| COPD | < 0.001 | | ✓ | | 1.04 |  |
| DM | 0.380 | |  | |  |  |
| CKD | 0.015 | |  | |  |  |
| **Medication** |  | |  | |  |  |
| Anti-platelet | 0.002 | | ✓ | | 1.33 |  |
| Beta blockers | < 0.001 | | ✓ | | 1.25 |  |
| ACEI/ARB | < 0.001 | | ✓ | | 1.08 |  |
| Heparin | 0.013 | |  | |  |  |
| Statins | 0.002 | | ✓ | | 1.34 |  |
| MRA | < 0.001 | | ✓ | | 1.05 |  |
| Diuretics | < 0.001 | |  | |  |  |
| **Vital signs** |  | |  | |  |  |
| SBP | 0.170 | |  | |  |  |
| SpO₂ | 0.012 | |  | |  |  |
| **Lab data** |  | |  | |  |  |
| Hemoglobin | 0.117 | |  | |  |  |
| WBC | 0.063 | |  | |  |  |
| PLT | < 0.001 | | ✓ | | 1.13 |  |
| K⁺ | 0.288 | |  | |  |  |
| Na⁺ | 0.812 | |  | |  |  |
| Lactate | < 0.001 | | ✓ | | 1.21 |  |
| pH | 0.001 | |  | |  |  |
| TBIL | < 0.001 | | ✓ | | 1.13 |  |
| Glucose | < 0.001 | | ✓ | | 1.09 |  |
| Cr | < 0.001 | |  | |  |  |
| **Organ support** |  | |  | |  |  |
| CRRT | < 0.001 | |  | |  |  |
| Mechanical ventilation | < 0.001 | |  | |  |  |
| **Clinical scoring** |  | |  | |  |  |
| OASIS | < 0.001 | | ✓ | | 1.66 |  |
| SOFA | < 0.001 | | ✓ | | 2.00 |  |

BMI, Body Mass Index; CVA, Cerebrovascular Accident; AF, Atrial Fibrillation; HTN, Hypertension; HEP, hepatic disease; HF, Heart Failure; COPD, Chronic Obstructive Pulmonary Disease; DM, Diabetes Mellitus; CKD, Chronic Kidney Disease; ACEI/ARB, Angiotensin-Converting Enzyme Inhibitor/Angiotensin II Receptor Blocker; MRA, Mineralocorticoid Receptor Antagonist; SBP, Systolic Blood Pressure; SpO₂, Saturation of Peripheral Oxygen; WBC, White Blood Cell Count; PLT, Platelet Count; K⁺, Potassium; Na⁺, Sodium; TBIL, Total Bilirubin; Cr, Creatinine; CRRT, Continuous Renal Replacement Therapy; OASIS, Oxford Acute Severity of Illness Score; SOFA, Sequential Organ Failure Assessment.

Supplementary Table S3. Balance of Baseline Covariates Before and After Inverse Probability of Treatment Weighting (IPTW)

| **Characteristic** | **Before Weighting SMD** | | **After IPTW**  **SMD** |  |
| --- | --- | --- | --- | --- |
| **Demographics** |  |  | | |
| Age | 0.104 | | 0.042 |  |
| Gender (Male) | 0.081 | | 0.010 |  |
| BMI | 0.117 | | 0.024 |  |
| **Comorbidities** |  | |  |  |
| CVA | 0.017 | | 0.012 |  |
| AF | 0.035 | | 0.043 |  |
| HTN | 0.110 | | 0.018 |  |
| HEP | 0.034 | | 0.006 |  |
| HF | 0.109 | | 0.012 |  |
| COPD | 0.005 | | 0.012 |  |
| DM | 0.062 | | 0.008 |  |
| CKD | 0.040 | | 0.025 |  |
| **Medication** |  | |  |  |
| Anti-platelet | 0.107 | | 0.009 |  |
| Beta blockers | 0.112 | | 0.014 |  |
| ACEI/ARB | 0.144 | | 0.037 |  |
| Heparin | 0.233 | | 0.012 |  |
| Statins | 0.086 | | 0.010 |  |
| MRA | 0.035 | | 0.004 |  |
| Diuretics | 0.079 | | 0.007 |  |
| **Vital signs** |  | |  |  |
| SBP | 0.275 | | 0.052 |  |
| SpO₂ | 0.447 | | 0.020 |  |
| **Lab data** |  | |  |  |
| Hemoglobin | 0.585 | | 0.061 |  |
| WBC | 0.025 | | 0.011 |  |
| PLT | 0.474 | | 0.010 |  |
| K⁺ | 0.037 | | 0.022 |  |
| Na⁺ | 0.020 | | 0.014 |  |
| Lactate | 0.157 | | 0.011 |  |
| pH | 0.279 | | 0.022 |  |
| TBIL | 0.290 | | 0.024 |  |
| Glucose | 0.293 | | 0.014 |  |
| Cr | 0.172 | | 0.094 |  |
| **Organ support** |  | |  |  |
| CRRT | 0.057 | | 0.004 |  |
| Mechanical ventilation | 0.103 | | 0.012 |  |
| **Clinical scoring** |  | |  |  |
| OASIS | 0.173 | | 0.038 |  |
| SOFA | 0.417 | | 0.061 |  |

BMI, Body Mass Index; CVA, Cerebrovascular Accident; AF, Atrial Fibrillation; HTN, Hypertension; HEP, hepatic disease; HF, Heart Failure; COPD, Chronic Obstructive Pulmonary Disease; DM, Diabetes Mellitus; CKD, Chronic Kidney Disease; ACEI/ARB, Angiotensin-Converting Enzyme Inhibitor/Angiotensin II Receptor Blocker; MRA, Mineralocorticoid Receptor Antagonist; SBP, Systolic Blood Pressure; SpO₂, Saturation of Peripheral Oxygen; WBC, White Blood Cell Count; PLT, Platelet Count; K⁺, Potassium; Na⁺, Sodium; TBIL, Total Bilirubin; Cr, Creatinine; CRRT, Continuous Renal Replacement Therapy; OASIS, Oxford Acute Severity of Illness Score; SOFA, Sequential Organ Failure Assessment.

Supplementary Table S4: Association of initial laxative strategy with short and long-term mortality after PSM

| **Variables** | **Event** **n (%)** | **Model I** | | | **Model II** | |
| --- | --- | --- | --- | --- | --- | --- |
|  |  | **OR/HR (95%CI)** | **P** | **OR/HR (95%CI)** | | **P** |
| **ICU mortality (OR)** | | | | | | |
| Stimulant Laxatives | 81.00 (12.88%) | Ref | | Ref | | |
| Osmotic Laxatives | 81.00 (12.88%) | 1.00(0.70 - 1.44) | 0.999 | 0.80(0.44 - 1.45) | | 0.456 |
| Stool Softeners | 59.00 (9.38%) | 0.65(0.44 - 0.97) | 0.033 | 0.57(0.30 - 1.08) | | 0.084 |
| P for trend | - | 0.036 | | 0.086 | | |
| **In-hospital mortality (OR)** | | | | | | |
| Stimulant Laxatives | 111.00 (17.65%) | Ref | | Ref | | |
| Osmotic Laxatives | 96.00 (15.26%) | 0.81(0.59 - 1.13) | 0.212 | 0.52(0.30 - 0.89) | | 0.017 |
| Stool Softeners | 76.00 (12.08%) | 0.59(0.42 - 0.83) | 0.003 | 0.39(0.22 – 0.69) | | 0.001 |
| P for trend |  | 0.003 | | 0.001 | | |
| **28-day mortality (HR)** |  |  | |  | | |
| Stimulant Laxatives | 124.00 (19.71%) | Ref | | Ref | | |
| Osmotic Laxatives | 108.00 (17.17%) | 0.86(0.66 - 1.11) | 0.246 | 0.72(0.55 - 0.95) | | 0.019 |
| Stool Softeners | 77.00 (12.24%) | 0.60(0.45 – 0.79) | ＜0.001 | 0.55(0.40 - 0.76) | | ＜0.001 |
| P for trend |  | ＜0.001 | | ＜0.001 | | |
| **365-day mortality (HR)** |  |  | |  | | |
| Stimulant Laxatives | 204.00 (32.43%) | Ref | | Ref | | |
| Osmotic Laxatives | 186.00 (29.57%) | 0.90(0.74 - 1.09) | 0.282 | 0.81(0.65 - 1.00) | | 0.054 |
| Stool Softeners | 133.00 (21.14%) | 0.61(0.49 - 0.76) | ＜0.001 | 0.57(0.45 – 0.72) | | ＜0.001 |
| P for trend |  | ＜0.001 | | ＜0.001 | | |

Note: Model I: Unadjusted models. Model II: Adjusted for age; heart failure; chronic obstructive pulmonary disease; lactate, platelet count, total bilirubin, and glucose levels; SOFA and OASIS scores; and the use of diuretics, mineralocorticoid receptor antagonists, angiotensin-converting enzyme inhibitor/angiotensin II receptor blocker, beta-blockers, antiplatelet agents, and statins. The P for trend was calculated by treating the laxative categories as an ordinal variable. The stimulant laxative group served as the reference. Estimates are presented as Odds Ratios (ORs) for ICU and in-hospital mortality, and as Hazard Ratios (HRs) for 28-day and 365-day mortality.

Supplementary Table S5: Association of initial laxative strategy with with secondary clinical outcomes after PSM

| **Variables** | **Event** **n (%)** | **Model I** | | | **Model II** | |
| --- | --- | --- | --- | --- | --- | --- |
|  |  | **OR (95%CI)** | **P** | **OR (95%CI)** | | **P** |
| **Malignant arrhythmia** | | | | | | |
| Stimulant Laxatives | 70.00 (11.13%) | Ref | | Ref | | |
| Osmotic Laxatives | 68.00(10.81%) | 0.97(0.68 - 1.38) | 0.857 | 0.93(0.64 - 1.36) | | 0.704 |
| Stool Softeners | 74.00 (11.76%) | 1.06(0.75 - 1.51) | 0.723 | 1.08(0.74 - 1.57) | | 0.684 |
| P for trend |  | 0.721 | | 0.684 | | |
| **Cardiogenic shock** | | | | | | |
| Stimulant Laxatives | 107.00 (17.01%) | Ref | | Ref | | |
| Osmotic Laxatives | 84.00 (13.35%) | 0.74(0.54 - 1.02) | 0.065 | 0.68(0.47 - 1.00) | | 0.048 |
| Stool Softeners | 86.00 (13.67%) | 0.76(0.56 - 1.05) | 0.094 | 0.75(0.52 – 1.08) | | 0.125 |
| P for trend |  | 0.088 | | 0.111 | | |
| **Bowel sound recovery** |  |  |  |  | |  |
| Stimulant Laxatives | 178.00 (28.30%) | Ref | | Ref | | |
| Osmotic Laxatives | 245.00 (38.95%) | 1.77(1.37 - 2.30) | ＜0.001 | 1.72(1.31 - 2.26) | | ＜0.001 |
| Stool Softeners | 262.00 (41.65%) | 2.02(1.56 - 2.62) | ＜0.001 | 2.13(1.62 - 2.82) | | ＜0.001 |
| P for trend |  | ＜0.001 | | ＜0.001 | | |
| **Delirium** |  |  |  |  | |  |
| Stimulant Laxatives | 188.00 (29.89%) | Ref | | Ref | | |
| Osmotic Laxatives | 161.00 (25.60%) | 0.79(0.61 - 1.03) | 0.077 | 0.74(0.55 - 0.98) | | 0.039 |
| Stool Softeners | 122.00 (19.40%) | 0.54(0.41 - 0.71) | ＜0.001 | 0.51(0.38 – 0.69) | | ＜0.001 |
| P for trend |  | ＜0.001 | | ＜0.001 | | |

Note: Model I: Unadjusted models. Model II: Adjusted for age; heart failure; chronic obstructive pulmonary disease; lactate, platelet count, total bilirubin, and glucose levels; SOFA and OASIS scores; and the use of diuretics, mineralocorticoid receptor antagonists, angiotensin-converting enzyme inhibitor/angiotensin II receptor blocker, beta-blockers, antiplatelet agents, and statins. The P for trend was calculated by treating the laxative categories as an ordinal variable. The stimulant laxative group served as the reference.

Supplementary Table S6: Association of initial laxative strategy with short and long-term mortality after IPTW.

| **Variables** | **Event** **n (%)** | **Model I** | | | **Model II** | |
| --- | --- | --- | --- | --- | --- | --- |
|  |  | **OR/HR (95%CI)** | **P** | **OR/HR (95%CI)** | | **P** |
| **ICU mortality (OR)** | | | | | | |
| Stimulant Laxatives | 204.00 (11.45%) | Ref | | Ref | | |
| Osmotic Laxatives | 93.00 (12.09%) | 1.08(0.94 - 1.25) | 0.279 | 1.02(0.85 - 1.23) | | 0.830 |
| Stool Softeners | 65.00 (6.14%) | 0.79(0.68 - 0.92) | 0.003 | 0.73(0.60 - 0.89) | | 0.002 |
| P for trend | - | 0.003 | | 0.002 | | |
| **In-hospital mortality (OR)** | | | | | | |
| Stimulant Laxatives | 293.00 (16.44%) | Ref | | Ref | | |
| Osmotic Laxatives | 114.00 (14.82%) | 0.88(0.77 - 1.00) | 0.050 | 0.74(0.63 - 0.88) | | ＜0.001 |
| Stool Softeners | 87.00 (8.22%) | 0.68(0.59 - 0.77) | ＜0.001 | 0.58(0.49 – 0.69) | | ＜0.001 |
| P for trend |  | ＜0.001 | | ＜0.001 | | |
| **28-day mortality (HR)** |  |  | |  | | |
| Stimulant Laxatives | 346.00 (19.42%) | Ref | | Ref | | |
| Osmotic Laxatives | 124.00 (16.12%) | 0.90(0.71 - 1.13) | 0.356 | 0.81(0.63 - 1.04) | | 0.097 |
| Stool Softeners | 92.00 (8.69%) | 0.62(0.48 – 0.81) | ＜0.001 | 0.59(0.44 - 0.78) | | ＜0.001 |
| P for trend |  | ＜0.001 | | ＜0.001 | | |
| **365-day mortality (HR)** |  |  | |  | | |
| Stimulant Laxatives | 605.00 (33.95%) | Ref | | Ref | | |
| Osmotic Laxatives | 217.00 (28.22%) | 0.90(0.76 - 1.08) | 0.286 | 0.87(0.72 - 1.04) | | 0.120 |
| Stool Softeners | 174.00 (16.43%) | 0.66(0.55 - 0.80) | ＜0.001 | 0.59(0.48 – 0.73) | | ＜0.001 |
| P for trend |  | ＜0.001 | | ＜0.001 | | |

Note: Model I: Unadjusted models. Model II: Adjusted for age; heart failure; chronic obstructive pulmonary disease; lactate, platelet count, total bilirubin, and glucose levels; SOFA and OASIS scores; and the use of diuretics, mineralocorticoid receptor antagonists, angiotensin-converting enzyme inhibitor/angiotensin II receptor blocker, beta-blockers, antiplatelet agents, and statins. The P for trend was calculated by treating the laxative categories as an ordinal variable. The stimulant laxative group served as the reference. Estimates are presented as Odds Ratios (ORs) for ICU and in-hospital mortality, and as Hazard Ratios (HRs) for 28-day and 365-day mortality.

Supplementary Table S7: Association of initial laxative strategy with with secondary clinical outcomes after IPTW.

| **Variables** | **Event** **n (%)** | **Model I** | | | **Model II** | |
| --- | --- | --- | --- | --- | --- | --- |
|  |  | **OR (95%CI)** | **P** | **OR (95%CI)** | | **P** |
| **Malignant arrhythmia** | | | | | | |
| Stimulant Laxatives | 250.00 (14.03%) | Ref | | Ref | | |
| Osmotic Laxatives | 75.00 (9.75%) | 0.82(0.71 - 0.96) | 0.008 | 0.80(0.69 - 0.93) | | 0.004 |
| Stool Softeners | 91.00 (8.59%) | 0.82(0.71 - 0.95) | 0.009 | 0.81(0.70 - 0.94) | | 0.006 |
| P for trend | - | 0.008 | | 0.006 | | |
| **Cardiogenic shock** | | | | | | |
| Stimulant Laxatives | 343.00 (19.25%) | Ref | | Ref | | |
| Osmotic Laxatives | 95.00 (12.35%) | 0.77(0.67 - 0.87) | ＜0.001 | 0.69(0.59 - 0.79) | | ＜0.001 |
| Stool Softeners | 104.00 (9.82%) | 0.75(0.66 - 0.86) | ＜0.001 | 0.69(0.60 – 0.80) | | ＜0.001 |
| P for trend |  | ＜0.001 | | ＜0.001 | | |
| **Bowel sound recovery** |  |  |  |  | |  |
| Stimulant Laxatives | 322.00 (18.07%) | Ref | | Ref | | |
| Osmotic Laxatives | 330.00 (42.91%) | 1.70(1.54 - 1.89) | ＜0.001 | 1.76(1.58 - 1.96) | | ＜0.001 |
| Stool Softeners | 502.00 (47.40%) | 1.80(1.63 - 2.00) | ＜0.001 | 1.94(1.74 - 2.17) | | ＜0.001 |
| P for trend |  | ＜0.001 | | ＜0.001 | | |
| **Delirium** |  |  |  |  | |  |
| Stimulant Laxatives | 477.00 (26.77%) | Ref | | Ref | | |
| Osmotic Laxatives | 193.00 (25.10%) | 0.88(0.79 - 0.98) | 0.021 | 0.83(0.74 - 0.93) | | 0.001 |
| Stool Softeners | 176.00 (16.62%) | 0.69(0.61 - 0.77) | ＜0.001 | 0.63(0.56 – 0.71) | | ＜0.001 |
| P for trend |  | ＜0.001 | | ＜0.001 | | |

Note: Model I: Unadjusted models. Model II: Adjusted for age; heart failure; chronic obstructive pulmonary disease; lactate, platelet count, total bilirubin, and glucose levels; SOFA and OASIS scores; and the use of diuretics, mineralocorticoid receptor antagonists, angiotensin-converting enzyme inhibitor/angiotensin II receptor blocker, beta-blockers, antiplatelet agents, and statins. The P for trend was calculated by treating the laxative categories as an ordinal variable. The stimulant laxative group served as the reference.
